# Molecular Cloning, Expression Pattern and Polymorphisms of NADPH-Cytochrome P450 Reductase in the Bird Cherry-Oat Aphid *Rhopalosiphum padi* (L.)

**DOI:** 10.1371/journal.pone.0154633

**Published:** 2016-04-28

**Authors:** Kang Wang, Xiong Peng, Yayun Zuo, Yuting Li, Maohua Chen

**Affiliations:** 1 College of Plant Protection, Northwest A&F University, Yangling, China; 2 Key Laboratory of Crop Pest Integrated Pest Management on the Loess Plateau of Ministry of Agriculture, Yangling, China; CNRS, FRANCE

## Abstract

NADPH–cytochrome P450 reductase (CPR) plays an important role in the cytochrome P450 (CYP)-mediated metabolism of endogenous and exogenous substrates. CPR has been found to be associated with insecticide metabolism and resistance in many insects. However, information regarding CPR in the bird cherry-oat aphid, *Rhopalosiphum padi*, is unavailable. In the current study, a full-length cDNA (2,476 bp) of *CPR* (*RpCPR*) encoding 681 amino acids was cloned from *R*. *padi*. Nucleotide sequence and deduced amino acid sequence analysis showed that RpCPR exhibits characteristics of classical CPRs and shares high identities with those of other insects, especially with the pea aphid, *Acyrthosiphon pisum*. The mRNA of *RpCPR* was expressed at all developmental stages, with the highest expression level found in the second instar and the lowest in adult. Expression levels of *RpCPR* in isoprocarb-resistant and imidacloprid-resistant strains were 3.74- and 3.53-fold higher, respectively, than that of a susceptible strain. *RpCPR* expression could also be induced by low concentrations (LC_30_) of isoprocarb and imidacloprid. Moreover, we sequenced the open reading frame (ORF) of *RpCPR* from 167 field samples collected in 11 geographical populations. Three hundred and thirty-four SNPs were detected, of which, 65 were found in more than two individuals. One hundred and ninety-four missense mutations were present in the amino acid sequence, of which, the P484S mutant had an allele frequency of 35.1%. The present results suggest that *RpCPR* may play an important role in the P450-mediated insecticide resistance of *R*. *padi* to isoprocarb and imidacloprid and possibly other insecticides. Meanwhile, *RpCPR*maintains high genetic diversity in natural individuals, which provides the possibility of studying potential correlations between variants and certain special physiological characters.

## Introduction

The bird cherry-oat aphid, *Rhopalosiphum padi* (L.), is one of the most important pests of wheat in temperate regions worldwide [1]. Aside from direct feeding damage, *R*. *padi* also transmits the barley yellow dwarf virus (BYDV), which causes economically important disease of small grains and leads to significantly reduced quality and yield [2–4]. To minimize economic losses, multiple types of insecticides are chronically and excessively used in aphid control in modern agriculture. Insecticide resistance or changes in insecticide susceptibility have been documented occasionally in *R*. *padi* and other wheat aphids in various parts of the world, and is a long-standing challenge for chemical pest management in aphids and other agricultural pests [5–9].

Many studies suggest that the cytochrome P450 monooxygenase (P450) system is involved in the detoxification of xenobiotics, as well as the metabolism of endogenous compounds. P450-mediated insecticide resistance has been characterized in many insects [10], including aphids [11]. P450s can degrade all classes of insecticides [10,12], and in this metabolic reaction, the iron atom in the heme group of P450 must accept two electrons from NADH/NADPH [13], and NADPH-cytochrome P450 reductase (CPR, also referred to as POR, CYPOR, OR, NCPR, and P450R) functions as an electron transporter (redox partner), accepting electrons from NADPH and transferring them to P450s [14–15]. In some P450s (CYP1A2, CYP3A4, etc.), cytochrome b_5_ may also act as a donator of a second electron [15,16]. In addition, numerous studies have shown that, although there is an extensive diversity of P450 isoforms, generally only one *CPR* gene exists in the genome of each creature, including insecta [15,17,18]. Therefore, the *CPR* gene is considered a vital part of P450-mediated insecticide resistance and is considered a novel target for the development of “smart” insecticides and synergists [19–20]. Inhibition of CPR reduces the activities of all microsomal P450 enzymes. Conditional deletion of CPR in the liver results in the inactivation of the hepatic P450 system [21]. In the bed bug *Cimex lectularius* and mosquito *Anopheles gambiae*, silencing of *CPR* resulted in increased susceptibility to pyrethroid insecticides in resistant populations [18,22]. RNA interference (RNAi) of *Nilaparvata lugens CPR* significantly reduced the transcription level and resulted in increased sensitivity to beta-cypermethrin and imidacloprid [23]. Additionally, directed RNAi of CPR of some insects significantly affected the biosynthesis of endogenous substances such as pheromone and cuticular hydrocarbon [24,25].

Another fascinating aspect of CPR is its numerous polymorphisms or/and mutations. Since the first report of CPR deficiency [26], many studies worldwide have described the varying phenotypes in humans; to date, over 2,000 single nucleotide polymorphisms (SNPs) have been described in human *CPR* genes, encompassing over 150 missense mutations that affect transcription, have also been identified in many syndromes [13,15]. However, few polymorphisms or/and mutations in insect *CPR* genes have been reported.

Genetic information regarding *CPR* has become available in several insect species since the first report of the cDNA and deduced protein sequence of CPR in the house fly, *Musca domestica* [27]. The *CPR* gene of the fruit fly, *Drosophila melanogaster* [28], silkworm, *Bombyx mori* [29] and cabbage armyworm, *Mamestra brassicae* [30] have been cloned and studied in terms of their involvement in odorant clearance and 20-hydroxyecdysone biosynthesis. Recently, the focus on insect *CPR* genes has shifted to insecticide resistance. Genetic studies of the mosquitos *Anopheles gambiae* [31] and *A*. *minimusm* [32], and bed bug, *C*. *lectularius* [18], among others, have shown that CPR is related to resistance to pyrethroid insecticides in public health pests. Meanwhile, more *CPR* genes were also characterized in agricultural pests such as the rice brown planthopper, *Nilaparvata lugens* [23] and diamondback moth, *P*. *xylostella* [33], and demonstrated to be associated with insecticide resistance. To our knowledge, there is no report concerning the sequences or functions of *CPR*, nor its polymorphisms or/and mutations in *R*. *padi*.

In the present study, *R*. *padi CPR* (*RpCPR*) was cloned and its expression pattern analyzed at various developmental stages. Its expression profiles in an isoprocarb resistant-strain and imidacloprid-resistant strain, in addition to the transcriptional response of *RpCPR* to the two insecticides in a susceptible strain (SS), were examined. Moreover, the gene polymorphisms or/and mutations in 167 natural individuals of 11 geographical populations in China were evaluated. These data may facilitate further study of the functions of CPR in P450-mediated isoprocarb resistance, imidacloprid resistance and other physiological mechanisms in *R*. *padi*. Moreover, the high genetic diversity of *RpCPR* in natural individuals provides the possibility of testing potential correlations between variants and several unique physiological characteristics.

## Materials and Methods

### Ethics Statement

No specific permissions were required for the described field studies for this wide spread agriculture pest. We confirm that the locations were not privately owned or protected in any way. The field studies did not involve endangered or protected species.

### Aphids

In this study, all *Rhopalosiphum padi* were reared at 23 ± 1°C, a photoperiod of L16:D8, and relative humidity of 60 ± 5%. Three bird cherry-oat aphid strains were used in this study. An insecticide-susceptible strain (SS) (LC_50_ values of 0.980 mg/L for imidacloprid and 1.032 mg/L for isoprocarb) first collected in Gansu Province, China in 2013 was maintained in the laboratory for more than three years without insecticide exposure. A imidacloprid-resistant strain (IM-R) and a isoprocarb-resistant strain (IS-R) was generated after continuous treatment with exposure to the respective two insecticides, which regularly kill 40% -70% of aphids, every 15 days. The IM-R strain showed an LC_50_ value of 21.3 mg /L for imidacloprid, with a resistance ratio of 21.7-fold, and the IS-R strain displayed a 32.4-fold increase in resistance compared to the SS strain, with an LC_50_ value of 33.4 mg/L for isoprocarb.

To detect RpCPR polymorphisms and mutations, we collected *R*. *padi* from 11 geographical populations in various wheat production areas of China (Table 1). All samples were collected from wheat. Each population was collected from at least eight collection points in a wheat field, and five individuals were obtained in each collection point. The distance between any two points was at least 30 m.

**Table 1:** Sampling information and population statistics for *R*. *padi* investigated using *RpCPR*.

Province	Region	Population code	*N*	*H*	*Hd*	*S*	*Pi*
Anhui	Chuzhou	AHCZ	18	18	1.000	39	0.00292
Gansu	Lanzhou	GSLZ	16	15	0.992	46	0.00375
Guizhou	Guiyang	GZGY	16	11	0.958	48	0.00463
Shaanxi	Hanzhong	SXHZ	14	13	0.989	33	0.00272
	Xianyang	SXXY	16	13	0.975	29	0.00279
Hebei	Baoding	HBBD	8	8	1.000	28	0.00400
Shandong	Heze	SDHZ	17	15	0.985	39	0.00297
Shanxi	Linfen	SXLF	16	11	0.942	27	0.00266
Hubei	Wuhan	HBWH	19	19	1.000	55	0.00357
Henan	Nanyang	HNNY	17	17	1.000	43	0.00309
Chongqing	Baibei	CQBB	10	10	1.000	22	0.00258

*N*, number of aphids successfully genotyped; *H*, number of haplotypes; *Hd*, haplotype diversity; *S*, number of polymorphic sites; *Pi*, nucleotide diversity.

### RNA Isolation and cDNA Synthesis

Total RNA was extracted using TRIzol reagent (Invitrogen, Carlsbad, CA, USA) according to the manufacturer’s instructions, and was treated with DNase I (Takara, Kyoto, Japan). For real-time quantitative polymerase chain reaction (RT-qPCR) analysis, 2 μg of total RNA (500 μg/mL) were reverse-transcribed into single-stranded cDNA with the reaction mixture containing 2 μL Oligo (dT) (500 μg/mL) and 4 μL distilled water using an M-MLV Reverse Transcriptase cDNA Synthesis Kit (Promega, Madison, WI, USA) according to the manufacturer’s recommendations. For amplification of *RpCPR*, cDNA was synthesized using a SMARTer^™^ RACE cDNA Amplification Kit (Clontech, Mountain View, CA, USA). The cDNA synthesized was stored at -20°C.

### Cloning of *RpCPR*

The full-length cDNA of *RpCPR* was cloned by RT-PCR and rapid amplification of cDNA ends (RACE). Firstly, Specific primers (*CPR*-F and *CPR*-R) were designed from the nucleotide sequence of the *Acyrthosiphon pisum CPR* gene (accession no. XM_001945277.3). Based on the partial putative fragment of *RpCPR* obtained via primer pair *CPR*-F and *CPR*-R, gene-specific primes for 5′-RACE (*RpCPR* -5R) and 3′-RACE (*RpCPR* -3R1 and *RpCPR* -3R2) were designed to clone the of 5′and 3′ sequences of the gene. To confirm the full length of the *RpCPR* linked from the 5′-RACE and 3′-RACE results, a specific primer pair (*RpCPR*-CF and *RpCPR*-CR) was designed to amplify the full length of the gene. All the primers used is showed in Table 2. All PCR products were purified with a Wizard PCR Preps kit (Promega, Madison, WI, USA). The PCR products purified were cloned into pGEM-T easy vectors (Promega, Madison, WI, USA) and transformed into *Escherichia coli* DH5α competent cells. Five positive clones of each sample were randomly chosen for bidirectional sequencing on an Applied Biosystems 3730 automated sequencer (Applied Biosystems, Foster City, CA, USA).

**Table 2:** Primers used for cloning and expression analysis of *RpCPR* in *R*. *padi*.

Gene	Primer Name	Primer Function	Primer Sequence (5′-3′)
*RpCPR*	*CPR*-F	Fragment cloning	GAAGAGCCATTGATTAGTGC
	*CPR*-R	Fragment cloning	TAATGGGAGCAAACACTATC
	*RpCPR* -5R	5′-RACE	CCATAAAATACTACCAAACTACGCCCG
	*RpCPR* -3R1	3′-RACE	GTGAGGGAGACCCAACAGATAATGC
	*RpCPR* -3R2	3′-RACE	CCGATTTGGACCATTTATGTGAACTAC
	*RpCPR*-CF	Cloning full length	TAACGTCGTGTACCGTAAGC
	*RpCPR*-CR	Cloning full length	AAGCCCACATCTCTTCCATT
	*RpCPR*-qF	qRT-PCR	TAAGCCCGATTTGGACC
	*RpCPR*-qR	qRT-PCR	GCAACACCTTTATTGACACG
*β*-actin	actin-F	Housekeeping gene analysis	GCCCAATCCAAAAGAGGTAT
	actin-R	Housekeeping gene analysis	TCAAAGGTGCTTCCGTTAGT

### Sequence Analysis of RpCPR

The full-length cDNA of *RpCPR* was assembled using DNAMAN version 5.2 (Lynnon Biosoft, San Ramon, CA, USA), and its ORF and deduced amino acid sequence were determined using ORF Finder (http://www.ncbi.nlm.nih.gov/gorf/gorf.html). The theoretical isoelectric point (pI) and molecular weight (MW) of RpCPR were calculated using ExPASy (www.expasy.org/tools/protparam.html). Putative transmembrane domains and signal peptides were predicted with TMHMM (www.cbs.dtu.dk/services/TMHMM-2.0/) and signalP (www.cbs.dtu.dk/services/SignalP/), respectively. The binding domains and catalytic residues were predicted by Conserved Domain Search (www.ncbi.nlm.nih.gov/Structure/cdd/cdd.shtml/). Sequence identification and similarities were analyzed using BLAST (blast.ncbi.nlm.nih.gov/blas). Amino acid sequences of RpCPR and orthologs from other insect species were aligned using ClustalW2 (http://www.ebi.ac.uk/Tools/msa/clustalw2/), and a phylogenetic tree was constructed by the neighbor-joining method with bootstrap test of 1,000 replicates using MEGA 5.0 software [34].

### Qualitative Real-Time PCR (qPCR) Analysis

Primers for RT-qPCR were designed using Primer 5 and are listed in Table 2. *β-actin* was used as the house-keeping gene in the analysis [35].The specificity of primer pairs (*RpCPR*-qF and *RpCPR*-qR, actin-F and actin-R) was tested and confirmed by sequencing basing on preliminary experiment (data not shown). RT-qPCR was performed on a LightCycler Nano System (Roche, Mannheim, Germany) with FastStart Essential DNA Green Master (Roche, Mannheim, Germany) in accordance with the manufacturer’s instructions. Using 20-fold-diluted cDNA as templates, all reactions were performed in a 20 μL final volume including 10 μL FastStart Essential DNA Green Master, 0.8 μL each specific primer (Table 2), 2 μL cDNA template, and 6.4 μL RNase-free water. The reaction was performed at 95°C for 3 min, followed by 40 cycles of 10 s at 95°C, 20 s at 58°C and 20 s at 72°C; a final melt-curve step was included post-PCR (ramping from 55°C to 95°C by 0.5°C every 5 s) to check for nonspecific amplification. Each reaction was performed in triplicate, after which the average threshold cycle (Ct) per sample was calculated. The *β-actin* gene of *R*. *padi* was used as the internal control gene to normalize the target gene expression levels. The relative expression of genes was calculated using 2^−ΔΔCt^ method. Three biological replicates were run for each experiment.

### *RpCPR* Expression in Different Development Stages and Different Strains

To investigate the expression pattern of *RpCPR* at various developmental stages, the relative transcript levels if the gene in all the five developmental stages (1^st^, 2^nd^, 3^rd^ and 4^th^ instar, and adult) of the *R*. *padi* were investigated by RT-qPCR. Total RNA was isolated from individuals (5 mg) of each developmental stages of SS strain. The aforementioned methods for RNA extraction and qPCR were used.

The relative expression level of *RpCPR* in IS-R, IM-R and SS strains was analyzed using qRT-PCR. Total RNA was isolated from individuals (5 mg) of each strain. The methods for RNA extraction and qPCR were as described above.

### *RpCPR* Expression after Exposure to Isoprocarb and imidacloprid

Isoprocarb and imidacloprid used in the study were of technical grade. The neonicotinoid, imidacloprid (95% purity), was provided by Jiangsu Changlong Chemical Co., Ltd., China. The carbamate, isoprocarb (95% purity), was provided by Anhui Huaxing Chemical Industry Co., Ltd., China.

Standard stock solutions (10 g/L) of each insecticide were prepared in acetone. Then, stock solutions were further diluted to the LC_30_ (0.567 mg/L for isoprocarb and 0.363 mg /L for imidacloprid) concentration using 0.1% Triton X-100 solution, with 0.0057% (v/v) acetone in isoprocarb solution and 0.0036% (v/v) acetone in imidacloprid solution, respectively. All solutions were stored in the dark at 4°C.

The previously reported leaf-dipping method was adopted for treatment [35,36]. Wheat leaves with apterous adult aphids (SS) were dipped into the aforementioned LC_30_ solutions which can killed around 30% of the aphids for 10 s, after which the leaves were taken out and residual solution droplets on the leaf were adsorbed using clean, dry filter paper. Leaves were dipped into 0.1% Triton X-100 only for use as controls. Three replicates of 50–60 aphids each were used for each chemical and control treatment. All aphids were maintained at a constant temperature of 23 ± 1°C and a photoperiod of 16:8 (L: D) h during and after treatment. Live aphids were collected at 3, 6, 12, and 24 h post-treatment, total RNA was isolated from individuals (5 mg) of the live aphids collected at each treatment, and the CPR expression level was analyzed. The methods for RNA extraction and qPCR were as described above.

### Polymorphism or/and Mutation Identification

RNA was isolated from apterous adult aphid from different geographical populations in China, and reverse-transcripted as described above. PCR amplification was performed using Takara LA-Taq DNA Polymerase (Takara Bio, Dalian, China) under cycling conditions of 5 min at 94°C followed by 30 cycles of 30 s at 94°C, 30 s at 55°C and 1 min at 72°C. PCR products were purified using a Wizard PCR Preps Kit (Promega, Madison, WI, USA), and the purified fragments were cloned into a pGEM-Teasy Vector (Promega, Madison, WI, USA) and transformed into *Escherichia coli* JM109. Finally, two recombinant plasmids with each insert were sequenced by Sangon Biotech (Shanghai, China) [37].

The nucleotide and acid amino sequences of the SS strain were used as the standard models. All nucleotide sequences were aligned with ClustalX, and SNPs and haplotypes were ascertained using DnaSP v5 [38] and MEGA 5.0 software.

### Statistical Analyses

Data analyses were carried out using SPSS statistics software (SPSS Inc., Chicago, IL, USA). For RT-qPCR results, all data were subjected to one-way analysis of variance (ANOVA) with the least significant difference test among multiple groups or analyzed by Student’s *t*-test (two-tailed paired t-test) between two groups and are expressed as the mean ± standard error (SE). The level of significance was set at *P*<0.05.

## Results

### Cloning and Sequence Analysis of *R*. *padi CPR*

A partial putative cDNA fragment of *R*. *padi CPR* (~650 bp) was amplified from apterous adult aphids (SS strain) by PCR using specific primers (CPR-F and CPR-R) designed based on the homologous gene sequence in *A*. *pisum*. BLAST analysis of the nucleotide sequence of the partial putative *RpCPR* cDNA showed that the sequence shared 94% nucleotide similarity with the corresponding sequence from the pea aphid. The complete cDNA of *RpCPR* has a 5′-untranslated region (5′-UTR) of 330 bp, a 3′-untranslated region (3′-UTR) of 103 bp, and an ORF of 2,046 bp, which encodes a protein of 681 amino acids. The pI and MW of *RpCPR* were predicted to be 5.27 and 77.11 kDa, respectively. The nucleotide sequence of *R*. *padi CPR* has been deposited into GenBank under accession number KU057505.

**Fig 1:**
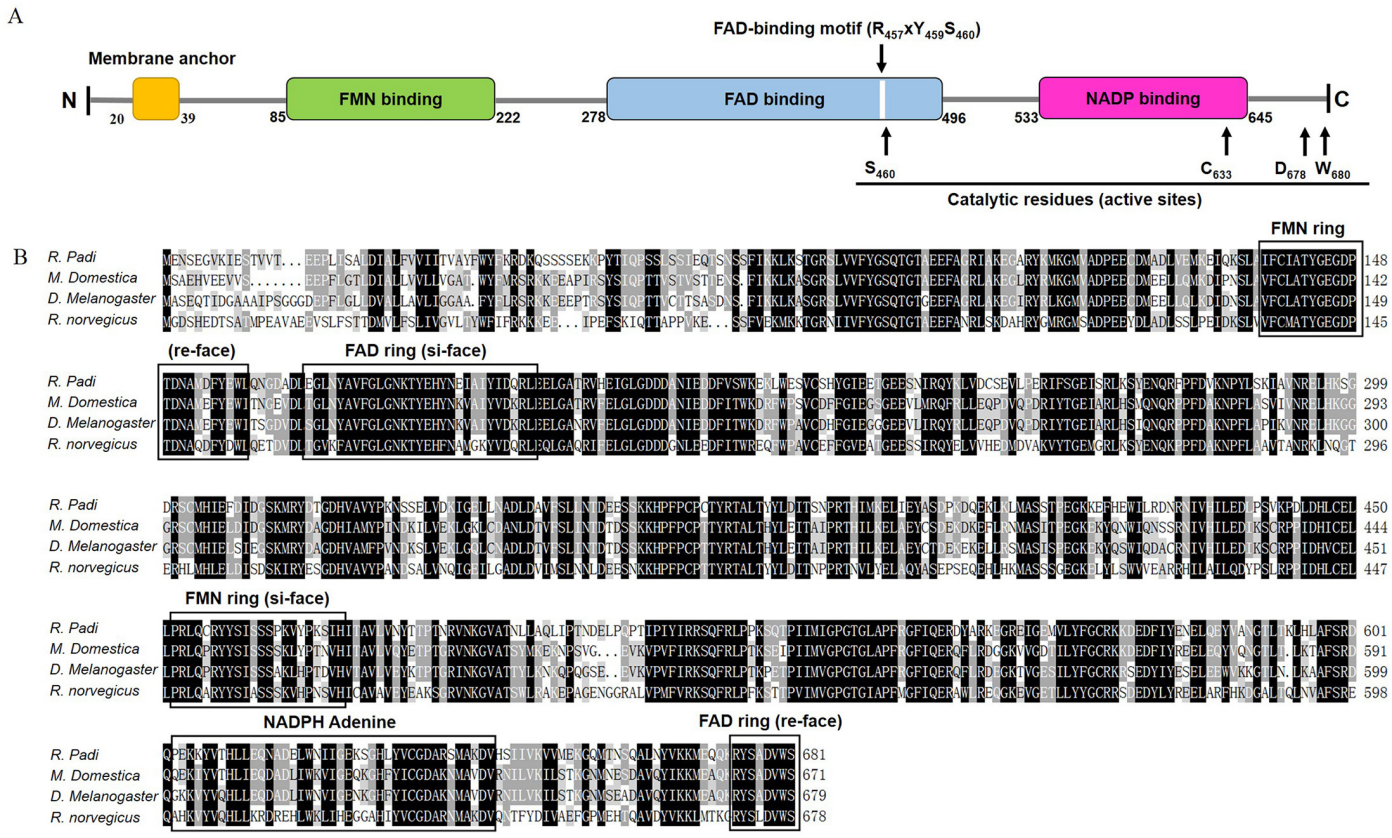
Sequence analysis of *R*. *padi* CPR. (A) Schematic drawing of RpCPR. Membrane anchor, conserved FMN-, FAD- and NADP-binding domains, FAD-binding motif (Arg_457_ x Tyr_459_ Ser_456_), and catalytic residues (Ser_460_, Cys_633_, Asp_678_ and Trp_680_) are shown. (B) Comparison of the deduced amino acid sequence of RpCPR with those of other CPRs deposited into GenBank. The species and accession numbers are as follows: *Musca domestica* (NP_001273818), *Drosophila melanogaster* (NP_477158) and *Rattus norvegicus* (NP_113764).

The RpCPR protein possesses several characteristic structural features. Multiple alignment of RpCPR and several other known CPRs (CRR of *M*. *domestica*, *D*. *melanogaster* and *Rattus norvegicus*) (Fig 1B) showed that RpCPR shares high identity with two other insect CPRs, and the three binding domains are conserved in the CPRs of insects and rats. No signal peptide was found within RpCPR, but the membrane anchor that facilitates the localization of RpCPR on the endoplasmic reticulum (ER) was identified at the N-terminus (Fig 1A). The amino acid residues arginine 457, tyrosine 459 and serine 460 constituted the consensus binding site of the flavin adenine dinucleotide (FAD)-binding motif, which is ubiquitous in the FAD-binding domain [39] (Fig 1A). Four catalytic residues (serine 460, cysteine 631, aspartic 676, and tryptophan 678) (Fig 1A) formed the active site, which has been demonstrated to be critical in the hydride transfer reaction [40,41].

### Phylogenetic Relationship between RpCPR and Other Insect CPRs

**Fig 2:**
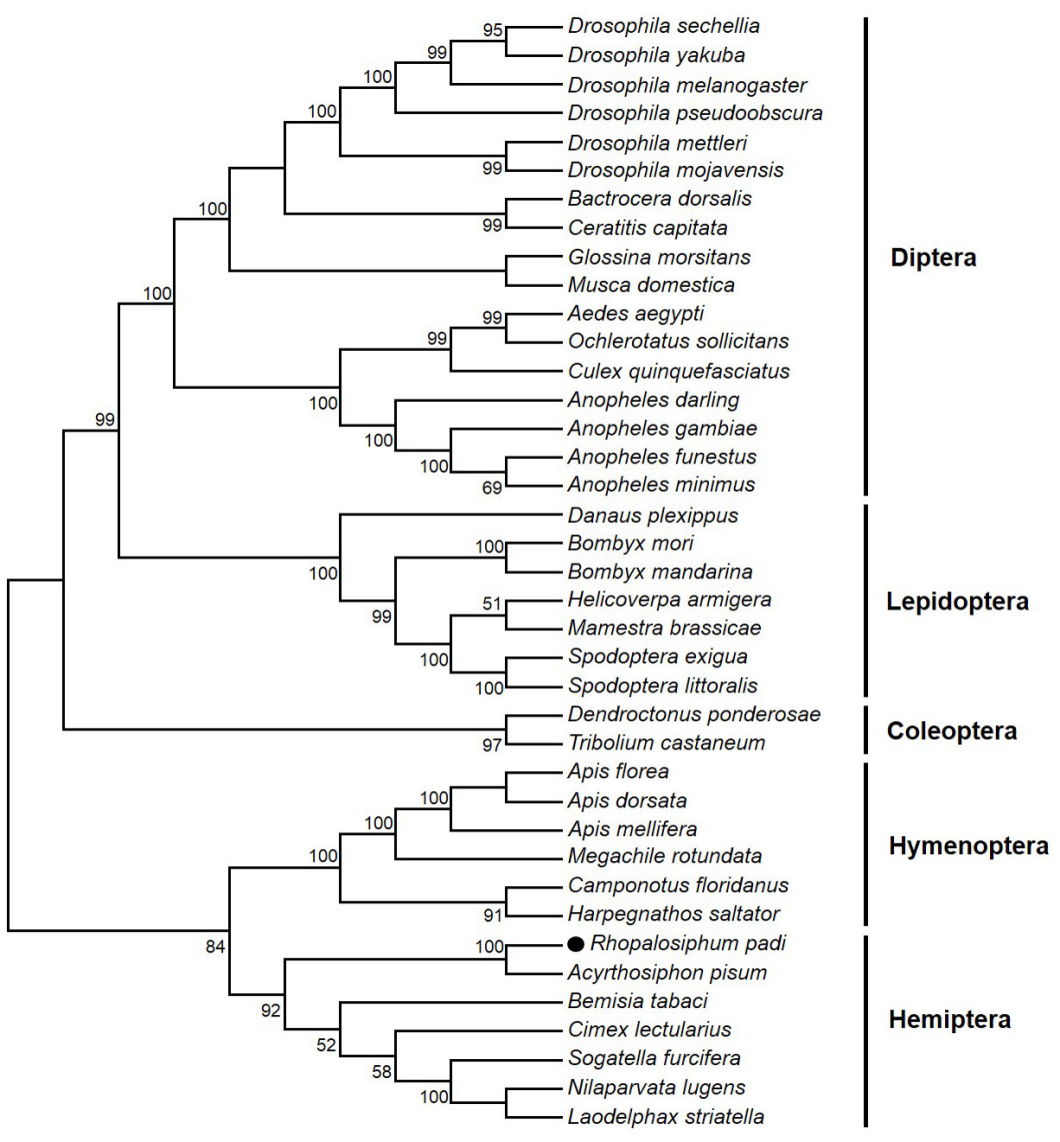
Phylogenetic tree of *R*. *padi* CPR with other insect CPRs. The neighbor-joining tree was generated using MEGA 5.0 software, and the phylogeny was tested by the bootstrap method with 1,000 replications. Bootstrap values >50% are shown. *R*. *padi* CPR is indicated by solid circles. The GenBank accession numbers of the sequences used were listed in supporting information S1 Table.

Phylogenetic analysis was performed using MEGA 5.0 software based on the deduced amino acid sequences of RpCPR and 38 other insect CPRs (S1 Table). The neighbor-joining phylogenetic tree showed that CPRs from the same insect order were clustered in the same branch (Fig 2). RpCPR and pea aphid *A*. *pisum* CPR were grouped together with strong bootstrap support (100%).

### Developmental Expression Profiles of *R*. *padi* CPR

**Fig 3:**
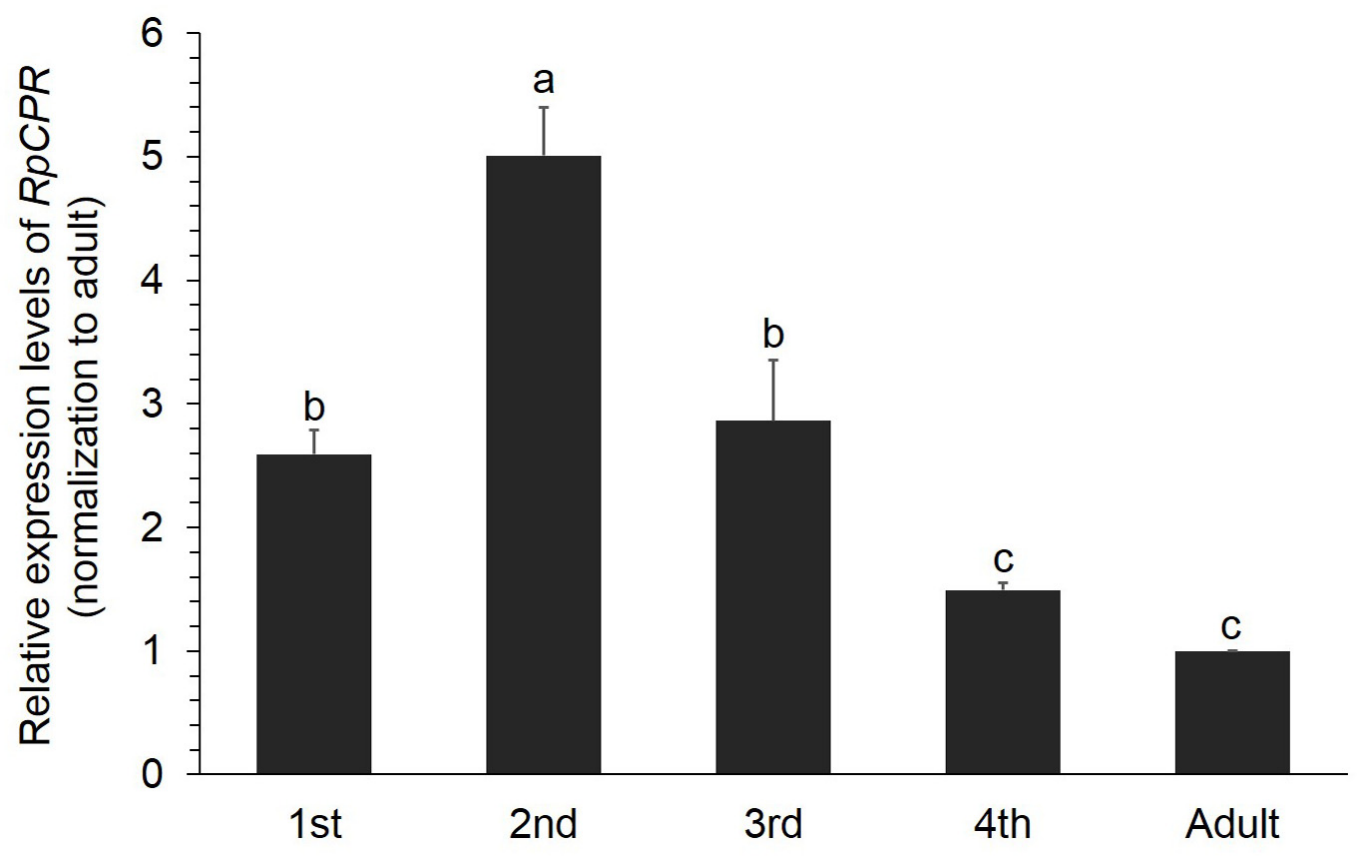
Relative expression of *R*. *padi* CPR at different developmental stages. Relative expression levels of *RpCPR* at different developmental stages were normalized to those in the adult. Data shown as the mean ± SE; different letters denoted a significant difference among samples (*P*<0.05, one-way ANOVA).

Insect P450s mediate a series of metabolic processes during the whole insect life cycle, and as an important electron transfer partner, the development-related expression profile of CPR could be a reflection of P450 activity [23]. *RpCPR* transcripts were detected at all developmental stages including the 1^st^, 2^nd^, 3^rd^, 4^th^ instar and adult (Fig 3), and the transcript level rapidly increased from the first to second instar, and then gradually decreased from the second instar to adult. The expression level of *RpCPR* in the second instar was 5.01-fold higher than in other stages (*P*< 0.05).

### Expression of *RpCPR* in the IS-R, IM-R and SS Strains

**Fig 4:**
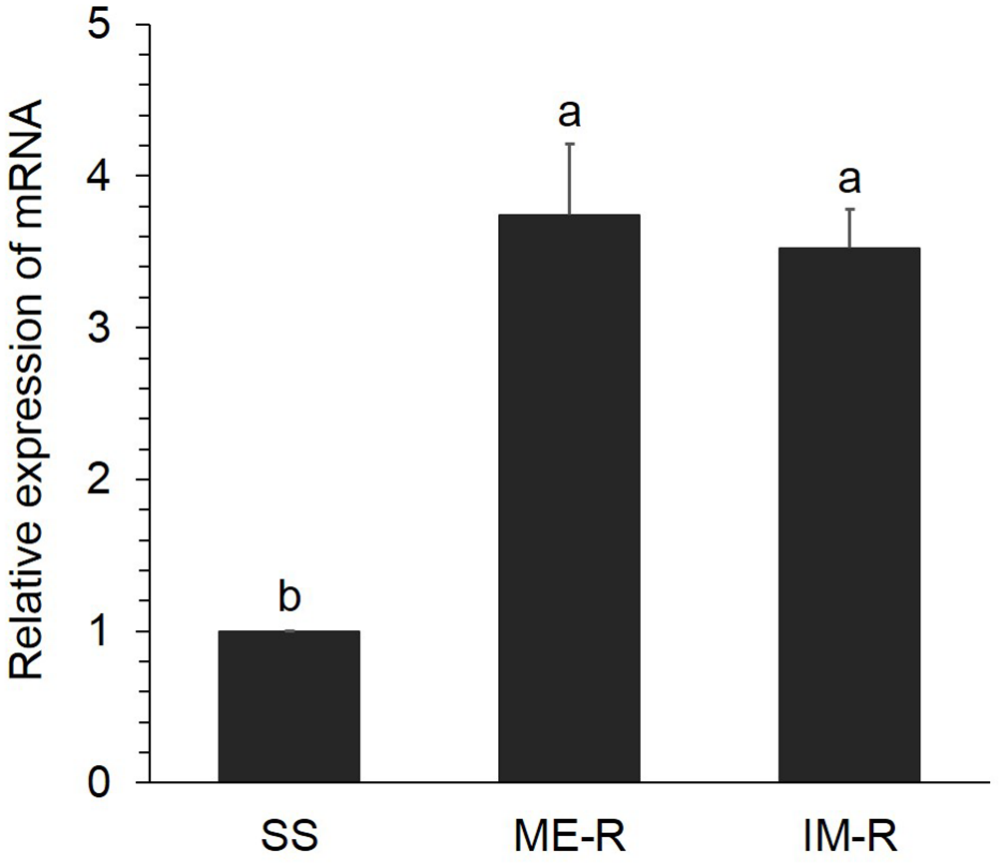
Relative expression levels of *RpCPR* in the susceptible strain (SS), the isoprocarb-resistant strain (IS-R) and the imidacloprid-resistant strain (IM-R). The expression level of *RpCPR* in SS was set to 1. Data shown as the mean ± SE; different letters denoted a significant difference among samples (*P*<0.05, one-way ANOVA).

The expression levels of *RpCPR* in the apterous adult aphids of the IS-R, IM-R and SS strains were analyzed. *RpCPR* expression was significantly higher in IS-R and IM-R than in SS (*P*<0.05), with increases of 3.74- and 3.52-fold, respectively, indicating its potential involvement in isoprocarb and imidacloprid resistance (Fig 4).

### Effect of Isoprocarb and Imidacloprid on *RpCPR* Expression

**Fig 5:**
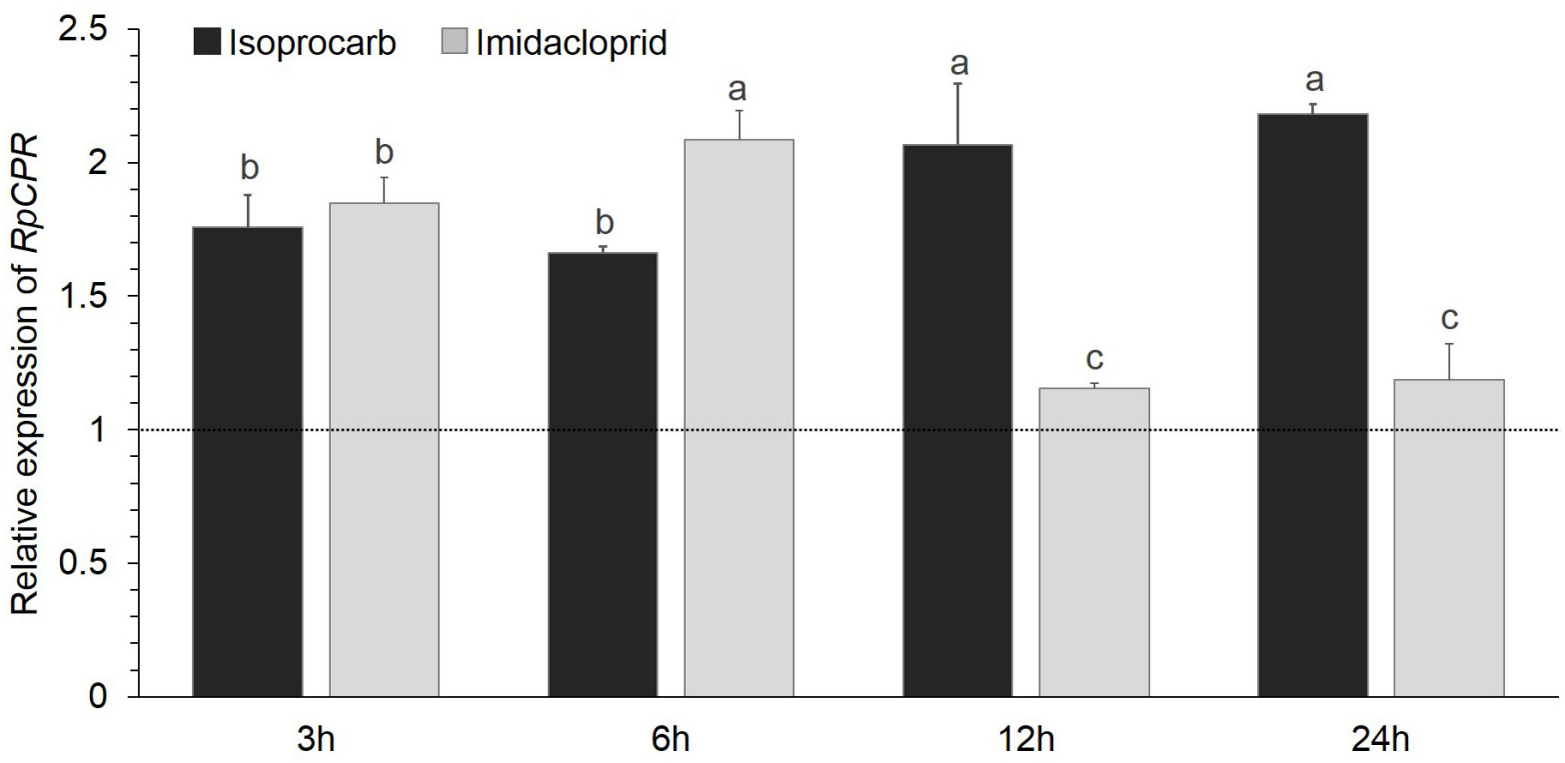
Relative expression levels of *RpCPR* in the SS strain treated with isoprocarb or/and imidacloprid. Data shown as the mean ± SE; different letters denoted a significant difference among samples (P<0.05, one-way ANOVA). The expression level of *RpCPR* in SS strain marked with a dash line at Y = 1.0.

Apterous adult aphids of the SS strain were exposed to the LC_30_ concentrations of isoprocarb and imidacloprid. *RpCPR* expression increased 1.66- to 2.18-fold after exposure to the isoprocarb (Fig 5). The *RpCPR* mRNA level was significantly increased at 3 h (1.85-fold) and 6 h (2.08-fold) after imidacloprid treatment, and then recovered to a normal level at 12 h (1.16-fold) and 24 h (1.19-fold) (Fig 5).

### *RpCPR* Polymorphisms in Field Samples

We sequenced the coding region of *RpCPR* from 167 individuals of 11 geographic populations in China (Table 1; GenBank accession numbers KU057506–KU057672). There were 146 haplotypes in the 167 individuals, and 334 distinct nucleotide variable sites were identified in the sequence (Table 1). Of these variable (polymorphic) sites, only 65 were detected in more than two individuals in the total population, and most were detected in one individual. The most common variants, A627T, G1362A, C1450T, and A1536T, were found in 31.7%, 35.3%, 35.3%, and 30.5% of the haplotypes, respectively.

**Fig 6:**
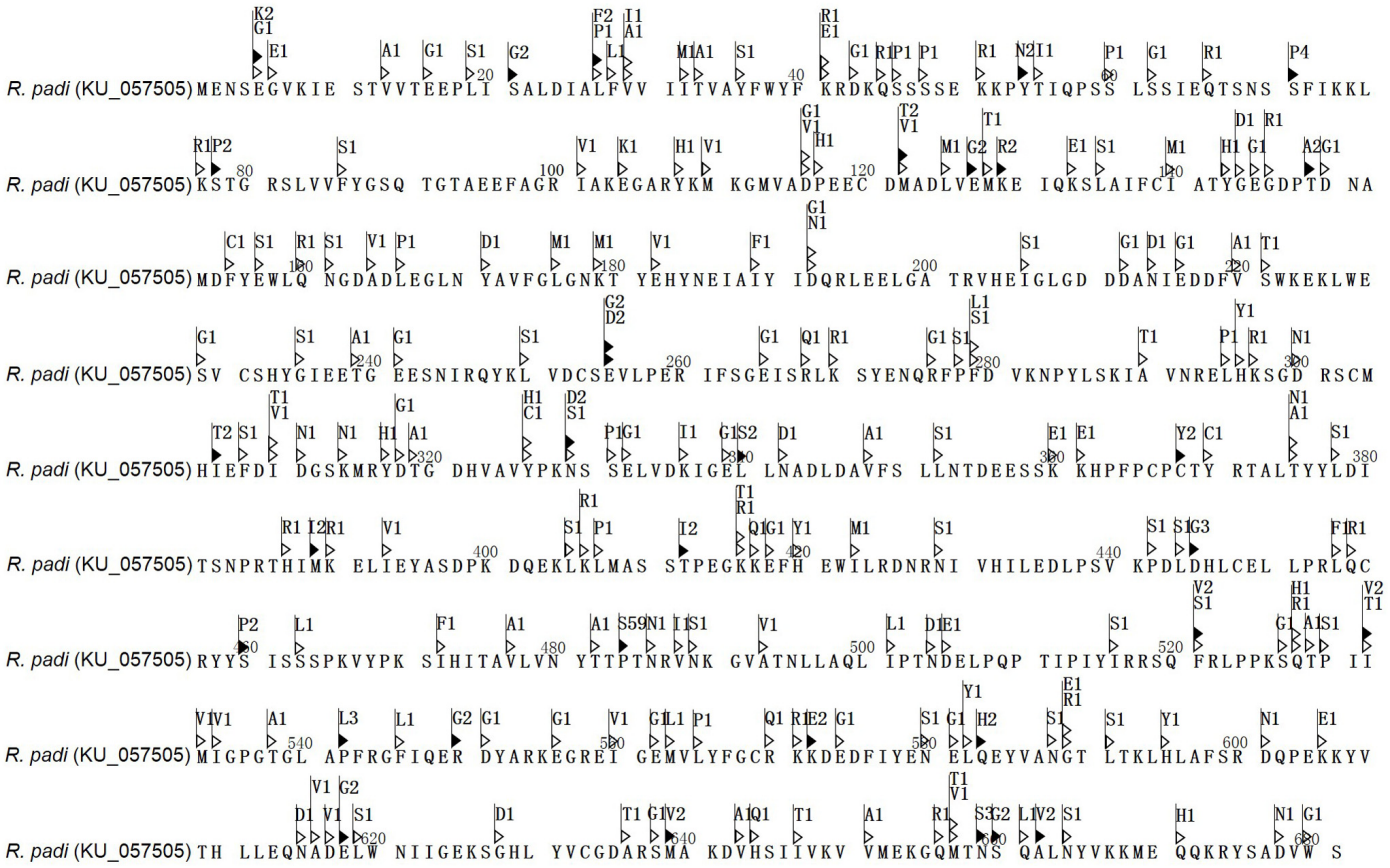
*R*. *padi* CPR protein sequence depicting missense mutations or/and polymorphisms. Mutation found only in one individual is indicated by a hollow triangle while polymorphic variants identified in more than two individuals are indicated by a solid triangle. The digital number after the mutated amino acids indicated the number of the aphid individuals with the mutation.

Missense mutations or/and polymorphisms in the amino acid sequence of RpCPR using the amino sequence of the SS strain as the standard are shown in Fig 6. In total, 194 missense mutations were found in the 167 aphid individuals, of which, 31 missense mutations belonged to parsimony-informative sites. Specifically, the amino acid sequence variant Pro484Ser, which resulted from the nucleotide variant C1450T, was found in 59 individuals from 11 geographic populations.

## Discussion

The cytochrome P450-mediated metabolic system is a major mechanism of insecticide resistance and the only system that can mediate resistance to all classes of insecticides [10]. In aphids, 83, 115 and 66 P450 genes were identified in pea aphid (*A*. *pisum*), green peach aphid (*Myzus persicae*) and cotton aphid (*Aphis gossypii*), respectively [42,43]. The *CYP6CY3* gene is associated with resistance to neonicotinoid insecticides in *M*. *persicae* [11, 44]. In the mono-oxygenation reaction, CPR is indispensable, as it functions as a unique electron transporter for almost all microsomal P450s [15]. Analysis of the function of insect CPR would enable further evaluation of the mechanism of insecticide resistance and facilitate the identification of new targets for insecticides.

In this study, the *RpCPR* gene was isolated from *R*. *padi*. Alignment analysis showed high identities of the deduced amino acid sequence of RpCPR with those of other insect CPRs. The phylogenetic analysis demonstrated that RpCPR and other hemipteran CPRs were clustered together within the branch for Hemiptera. Structure prediction indicated that RpCPR contains a membrane anchor in the N-terminal transmembrane region comprising 20 amino acid residues. Due to this hydrophobic segment, the remainder of CPR likely faces the cytoplasmic side of the membrane of the ER [45,46], which is important for electron transfer. Microsomal P450s are also inserted into the ER membrane by the N-terminal anchor sequence [13,47]. FMN (flavin mononucleotide)-binding, FAD-binding and NADPH (nicotinamide adenine dinucleotide phosphate)-binding domains were identified in the hydrophilic C-terminal catalytic domain of RpCPR, and alignment analysis with three classical CPRs demonstrated that these binding domains are highly conserved among the CPRs of insects and mammals (Fig 1). The FMN-binding domain of RpCPR comprises two conserved binding sites including the FMN ring (si-face) and FMN ring (re-face), which are critical for the interaction with the redox-partner binding site of P450s [48]. Electrons derived from NADPH in the form of two hydride ions are transferred to FAD, then to FMN. The electrons are then delivered one at a time to P450s or other acceptors [49–51]. The deduced amino acid sequence of *R*. *padi* CPR showed the highest identity to the CPR of *A*. *pisum*, which is consistent with the phylogenetic analysis results, showing that they cluster to the same monophyletic group (Fig 1). It is likely that these proteins perform similar physiological functions.

For the first time, we examined the expression profile of *CPR* mRNA in different developmental stages in an aphid species. *RpCPR* was expressed at various levels in *R*. *padi* during five life stages, likely due to different metabolic requirements at different developmental stages. *RpCPR* are involved in various demands of different P450s, which catalyze various endogenous biosynthesis and metabolic reactions. Insect Halloween P450 enzymes mediated the sequential hydroxylations of steroid precursors into the active ecdysteroid, 20-hydroxyecdysone [52,53]. CYP15A1, a P450 gene catalyzed epoxidation of methyl farnesoate to juvenile hormone in *Diploptera punctata* [54]. These two hormones governed the larval molting and metamorphosis. *CPR* genes of different insect species have diverse expression profiles throughout the life cycle. The expression levels of *N*. *lugens CPR* fluctuated during developmental stages; *NlCPR* transcription was highest in the first nymph and lowest in macropterous adults [23]. In *C*. *lectularius*, the *CPR* gene was ubiquitously expressed in all life stages, and its expression increased as immature stages developed into adults [18].

For detoxifying exogenous compounds such as insecticides in insects, the constitutively increased expression of P450 genes is thought to be directly linked to the degree of adaptation to the stress in question [55–58]. *RpCPR* was not only constitutively overexpressed in the imidacloprid- and isoprocarb-resistant strains but also significantly induced by both insecticides. Chen and Zhang (2015) found that *P*. *xylostella CPR* could be efficiently induced by a low dose of beta-cypermethrin, and was highly overexpressed in a field-collected beta-cypermethrin-resistant population, it was possible that the over-expression of CPR in resistant insects could effectively enhance P450 metabolism of insecticides [33]. Aside from CPRs, several insect P450 genes are overexpressed in insecticide-resistant strains and can be induced by chemical insecticides. The constitutive expression of *CYP6A1* in the house fly *M*. *domestica* is at least 10-fold higher in the resistant strain than the susceptible strain, and the gene is inducible by phenobarbital treatment of the flies [59]. Multiple P450 genes including *CYP6AA7*, *CYP9J40*, *CYP9J34*, and *CYP9M10* showed constitutive overexpression and permethrin induction in the insecticide-resistant mosquito, *Culex quinquefasciatus* [57]. Therefore, *Rp*CPR is likely involved in imidacloprid and isoprocarb resistance in *R*. *padi*. Further investigations that include the use of RNAi [18,28], transcriptomics [60,61] and metabolomics [62] are required to identify the underlying mechanism. CPR is indispensible for the function of P450s, and CPR inhibitors may enable P450-meditated insecticide resistance to be overcome in insect species.

Our analysis of *RpCPR* in 167 individuals from 11 geographic populations identified 334 SNPs, 194 of which belonged to nonsynonymous sites, which altered the encoded amino acid sequence. Most of these SNPs were found in only a single sample, while 65 SNPs were found in no fewer than two individuals of the total population. In particular, four SNPs (A627T, G1362A, C1450T, and A1536T) were present in ≥51 individuals, and the C1450T mutation resulted in the amino acid mutation Pro484Ser. More than 35% of individuals from different geographic populations harbored this missense mutation. Pro484Ser is located in the adenine ring (FAD-binding region), which is an important cofactor-binding region in the CPR family [45]. To date, few studies on the functions of CPR missense mutations in invertebrates have been conducted. Several synonymous mutations were detected in a fenvalerate-resistant strain of *H*. *armigera* [63]. Some site-directed mutations of *A*. *minimus* could enhance FAD or/and FMN binding by CRP, which suggests that CYP6AA3 mediates benzyloxyresorufin *O*-deakylation [64,65]. However, there are abundant reports of human CPR mutations. A total of over 2,000 SNPs, encompassing over 150 missense mutations, have been described in human CPR [15]. In human CPR, the Y181D mutation, found in patients with congenital adrenal hyperplasia, lacks FMN-binding activity [66]. The A287P, R457H, Y459H, and V492E mutations, which lie in the FAD-binding domain, greatly decrease FAD-binding affinity and disrupt both the 17α-hydroxylase and 17, 20 lyase activities of P450c17 [26,67]. Moreover, the overall 3D structures of some variants are similar to that of the wild type, while some subtle but significant differences exist, including local disruption of hydrogen bonding or/and salt bridging involving the FAD pyrophosphate moiety, leading to weaker FAD binding, an unstable protein and loss of catalytic activity [67,68]. A previous study on the common mutation in human CPR, A503V, which had an allele frequency of ~27% in 842 healthy unrelated humans, showed that the variant modestly but significantly decreased its catalytic activity, which may contribute to individual variations in drug response [69]. Additionally, in the CPR of *Saccharomyces cerevisiae*, T71A and D187A mutations in the FMN-binding site resulted in almost complete loss of function toward *CYP51* [48]. The polymorphism of human CPR could affect activity of some P450s or contributed to some human diseases or human variations to drug response, however, research about the effect of insect CPR gene mutations is rare. In this study, we report insect CPR polymorphisms in field samples, particularly some mutation sites, in several vital regions of RpCPR, indicating the genetic diversity of CPR in insects and a correlation between CPR mutations and insecticide metabolism or/and resistance. Further studies are needed to address the questions raised by our findings. For example, why there are so many mutations in RpCPR? Does insecticide selection pressure cause these mutations? PCR products were purified? Are abundant polymorphisms of CPR common in other insect species?

## Conclusions

The present study provides preliminary information on the sequence, phylogenetic relationships and expression pattern of CPR in the bird cherry-oat aphid, *R*. *padi*. The gene was overexpressed in the isoprocarb- and imidacloprid-resistant strains. Its expression was induced by sublethal concentrations of isoprocarb or/and imidacloprid. Our results indicate that *RpCPR* might be involved in resistance to isoprocarb or/and imidacloprid in *R*. *padi*. Furthermore, we examined the genetic diversity of *RpCPR* in several natural populations and described multiple mutation and polymorphism sites. Further studies are needed to investigate *RpCPR* variants with particular reference to the detoxification or/and activation of xenobiotics, as well as the metabolism of endogenous compounds. Indeed, further studies should assess the function of the *RpCPR* gene in individual P450-mediated detoxification pathways and other physiological processes in *R*. *padi*.

## Supporting Information

S1 Table Insect CPR in GenBank. (DOC)
