# Supplementary material for: Molecular Cloning, Expression Pattern and Polymorphisms of NADPH-Cytochrome P450 Reductase in the Bird Cherry-Oat Aphid Rhopalosiphum padi (L.)
Source: PLoS One. 2016 Apr 28;11(4):e0154633. doi: 10.1371/journal.pone.0154633 (PMC4849790; doi:10.1371/journal.pone.0154633)
Supplement: S1 Table — (DOC) [file pone.0154633.s001.doc]

Table S1. Insect CPR in GenBank

| **Order** | **Species** | **Accession numbers** |
| --- | --- | --- |
| Hemiptera | *Acyrthosiphon pisum* | XP_001945312 |
|  | *Cimex lectularius* | NP_001303631 |
|  | *Nilaparvata lugens* | AHB59865 |
|  | *Sogatella furcifera* | AHM93009 |
|  | *Laodelphax striatella* | AID55422 |
|  | *Bemisia tabaci* | AGT15701 |
| Lepidoptera | *Spodoptera exigua* | ADX95746 |
|  | *Danaus plexippus* | EHJ63867 |
|  | *Spodoptera littoralis* | AFP20584 |
|  | *Helicoverpa armigera* | ADK25060 |
|  | *Mamestra brassicae* | AAR26515 |
|  | *Bombyx mori* | NP_001104834 |
|  | *Bombyx mandarina* | ABJ97709 |
| Coleoptera | *Tribolium castaneum* | XP_971174 |
|  | *Dendroctonus ponderosae* | AFI45002 |
| Diptera | *Drosophila melanogaster* | NP_477158 |
|  | *Drosophila sechellia* | XP_002038080 |
|  | *Drosophila pseudoobscura* | XP_001355866 |
|  | *Drosophila yakuba* | XP_002088202 |
|  | *Drosophila mettleri* | AAB48964 |
|  | *Drosophila mojavensis* | XP_002002669 |
|  | *Ochlerotatus sollicitans* | ACL01092 |
|  | *Musca domestica* | NP_001273818 |
|  | *Bactrocera dorsalis* | XP_011214554 |
|  | *Ceratitis capitata* | XP_004523898 |
|  | *Glossina morsitans* | ADD19306 |
|  | *Aedes aegypti* | XP_001656715 |
|  | *Culex quinquefasciatus* | XP_001865801 |
|  | *Anopheles gambiae* | AAO24765 |
|  | *Anopheles funestus* | ABO77954 |
|  | *Anopheles minimus* | ABL75156 |
|  | *Anopheles darling* | ETN60137 |
| Hymenoptera | *Apis mellifera* | XP_006569767 |
|  | *Apis florea* | XP_012341484 |
|  | *Apis dorsata* | XP_006608682 |
|  | *Harpegnathos saltator* | EFN87403 |
|  | *Camponotus floridanus* | XP_011258273 |
|  | *Megachile rotundata* | XP_012145900 |
